# Progress in Flax Genome Assembly from Nanopore Sequencing Data

**DOI:** 10.3390/plants15010151

**Published:** 2026-01-04

**Authors:** Elena N. Pushkova, Alexander A. Arkhipov, Nadezhda L. Bolsheva, Tatiana A. Rozhmina, Alexander A. Zhuchenko, Elena V. Borkhert, Nikolai M. Barsukov, Gavriil A. Oleshnya, Alina V. Milovanova, Olesya D. Moskalenko, Fedor D. Kostromskoy, Elizaveta A. Ivankina, Ekaterina M. Dvorianinova, Daiana A. Krupskaya, Nataliya V. Melnikova, Alexey A. Dmitriev

**Affiliations:** 1 Engelhardt Institute of Molecular Biology, Russian Academy of Sciences, 119991 Moscow, Russia; pushkova18@gmail.com (E.N.P.); arkhipov.aleksandr2.0@gmail.com (A.A.A.); nlbolsheva@mail.ru (N.L.B.); sashai@inbox.ru (E.V.B.); keepter@yandex.ru (N.M.B.); ganyaole@gmail.com (G.A.O.); alina.milovanoff@gmail.com (A.V.M.); lesyamosk.bye-bye2003@yandex.ru (O.D.M.); fkostr@gmail.com (F.D.K.); sigova1567@gmail.com (E.A.I.); eugenevarenyuk@gmail.com (E.M.D.); zhernova.d@ya.ru (D.A.K.); mnv-4529264@yandex.ru (N.V.M.); 2 Moscow Center for Advanced Studies, 123592 Moscow, Russia; 3 Federal Research Center for Bast Fiber Crops, 172002 Torzhok, Russia; tatyana_rozhmina@mail.ru (T.A.R.); ecovilar@mail.ru (A.A.Z.); 4 All-Russian Horticultural Institute for Breeding, Agrotechnology and Nursery, 115598 Moscow, Russia; 5 Russian State Agrarian University—Moscow Timiryazev Agricultural Academy, 127434 Moscow, Russia

**Keywords:** flax, *Linum usitatissimum*, Nanopore, long-read sequencing, genome assembly, telomere-to-telomere, Hifiasm

## Abstract

In recent years, the quality of genome assemblies has notably improved, primarily due to advances in third-generation sequencing technologies and bioinformatics tools. In the present study, we obtained genome assemblies for two flax (*Linum usitatissimum* L.) varieties, K-3018 and Svyatogor, using Oxford Nanopore Technologies (ONT) simplex R10.4.1 data and the Hifiasm algorithm optimized for ONT reads. The K-3018 genome assembly was 491.1 Mb and consisted of thirteen full-length chromosomes and two one-gap chromosomes. The Svyatogor genome assembly was 497.8 Mb and consisted of twelve full-length chromosomes and three one-gap chromosomes. All chromosomes had telomeric repeats at their ends for both varieties. Hi-C contact maps and Illumina genomic data supported the accuracy of the obtained assemblies. The K-3018 and Svyatogor genome assemblies surpassed the quality of the best currently available flax genome assembly of variety T397, which serves as a reference for *L. usitatissimum* in the NCBI Genome database. Comparative analysis revealed that the flax genomes are generally quite similar at the chromosome level, with only a few large-scale differences. Thus, two near-T2T (telomere-to-telomere) flax genomes were assembled from the ONT simplex R10.4.1 reads using Hifiasm ONT without involving Pacific Biosciences (PacBio) HiFi or ultra-long ONT reads as well as optical maps. High-quality flax genomes are essential for improving the efficiency of genetic research, evaluating genetic diversity at the whole-genome level, and developing breeding and genome editing approaches of this valuable multipurpose crop.

## 1. Introduction

Over the past 25 years, significant progress has been made in plant genome sequencing, resulting in genome assemblies for numerous crop species [1]. In recent years, the quality of these assemblies has notably improved, primarily due to the advances in third-generation sequencing technologies, which can produce long reads spanning tens or hundreds of kilobases (kb), as well as improvements in bioinformatics tools for genome assembly [1,2,3,4]. There are two main third-generation sequencing platforms: Pacific Biosciences (PacBio), which produces high-accuracy HiFi reads, and Oxford Nanopore Technologies (ONT), which produces longer but less accurate reads. Various approaches and tools have also been developed to obtain high-quality genome assemblies from long reads [5,6,7].

High-quality plant genomes are used in many research areas, including functional genomics, evolutionary and phylogenetic studies, breeding, and genome editing [8,9,10,11,12,13,14]. The quality of genome assemblies is improving, and telomere-to-telomere (T2T) assembly is becoming the new standard in genomics for important crops [15].

Flax (*Linum usitatissimum* L.) is a multipurpose self-pollinated agricultural crop cultivated for its seeds and stems. The seeds are primarily used for pharmaceuticals and functional foods due to their high content of health-promoting lignans and omega-3 fatty acids, as well as for technical purposes [16,17,18,19,20,21]. The history of sequencing the flax genome (2n = 30) spans more than 10 years, progressing from an assembly of ~300 Mb consisting of multiple contigs to near-T2T-level genome assemblies of ~500 Mb [22,23,24,25,26,27,28,29,30,31]. In 2024, we assembled the 489 Mb genome of the variety K-3018 (NCBI Genome, GCA_048169125.1), which belongs to *L. usitatissimum* L. var. intermedia Vav. et Ell. and is considered linseed by the direction of its use. The assembly consisted of 22 large contigs. Eight full chromosomes were assembled from telomere to telomere. Five chromosomes were represented by two contigs each and two chromosomes were represented by three contigs each. All assembled chromosomes contained telomeric repeats at their ends [29]. Later, Lu et al. [30] stated that they assembled the gapless flax genome using ultra-long (UL) ONT and PacBio HiFi data. This 483 Mb genome contained telomeres at the ends of all 15 chromosomes. Unfortunately, this assembly, which was deposited to the China National Center for Bioinformation, will not be available until 30 June 2026 (CNCB, PRJCA037526, https://ngdc.cncb.ac.cn/gwh/Assembly/92583/show, accessed on 1 November 2025). Then, the 495 Mb flax genome of Indian linseed variety T397 was published by Yadav et al. It was primarily assembled from ONT and PacBio HiFi data and scaffolded into 20 super-scaffolds using optical mapping. Thirteen of the super-scaffolds corresponded to full-length chromosomes. The resulting 15-chromosome assembly had five gaps and 29 out of 30 telomeres were identified (NCBI Genome, GCA_051167515.1) [31].

Since we published our work on assembling the K-3018 flax genome, the Hifiasm algorithm optimized for ONT reads has emerged. This allowed high-quality genome assemblies to be produced using ONT sequencing data better than ever. The aim of our current study was to demonstrate progress in the flax genome assembly from ONT data by improving the quality of the variety K-3018 assembly and generating a genome assembly of the valuable dual-purpose (for seeds and fiber) flax variety Svyatogor (*L. usitatissimum* var. intermedia). Individual plants of varieties K-3018 and Svyatogor were previously selected by us for flax pan-genomic studies on the basis of genotyping results [32].

## 2. Results and Discussion

### 2.1. Genome of the Variety K-3018

**Figure 1:**
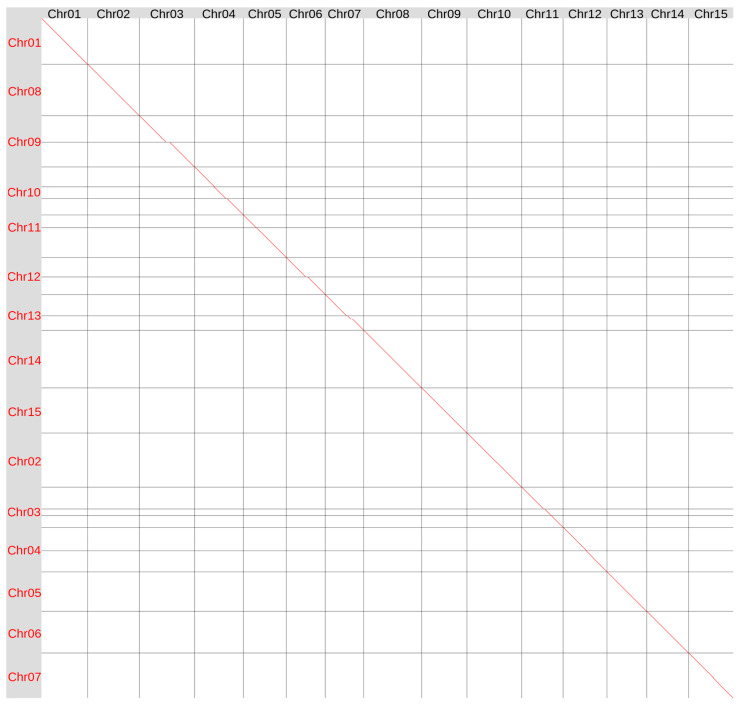
Global alignment of the genome assemblies of flax variety K-3018 obtained using Hifiasm v.0.19.9 (v1, Y axis) and Hifiasm ONT v.0.25.0 (v2, X axis). Red lines and dots–forward orientation, blue lines and dots–reverse orientation (no blue lines or dots in this alignment).

Previously, we sequenced the genome of variety K-3018 on the ONT platform and generated 57.7 Gb of ONT data corresponding to 115× coverage for a 500 Mb genome. After filtering for a minimum read length of 10 kb, 83× coverage remained. The genome was assembled using Hifiasm UL (v.0.19.9-r616) in double-graph mode using ONT reads corrected with the Herro algorithm integrated into the Dorado basecalling tool and deposited to NCBI Genome–GCA_048169125.1 [29]. In the present study, we reassembled the genome of variety K-3018 using the Hifiasm ONT algorithm (v.0.25.0-r726), which is optimized for ONT reads. We obtained the assembly of 491.1 Mb comprising 17 contigs, including 13 full-length chromosomes and two chromosomes split into two contigs each. Using the Hi-C data generated in this study, we successfully merged fragmented chromosomes 7 and 11 (Supplementary Figure S1). Telomeric repeats were identified at the ends of all 15 chromosomes (Supplementary Figure S2). Therefore, the Hifiasm ONT algorithm enabled us to significantly improve the contiguity of the K-3018 genome assembly, leaving only two gaps (versus nine in the previous version of the K-3018 genome assembly). Global genome alignment showed the similarity between the version 1 (v1) and version 2 (v2) assemblies of the K-3018 genome (Figure 1).

In the K-3018 v2 assembly, the chromosomes were named according to the chromosome numbers of the first flax genome assembly of the variety CDC Bethune (NCBI Genome, GCA_000224295.2) [23], following the consensus genetic map of flax [33]. In contrast, in the YY5, K-3018 v1, and T397 genome assemblies, the chromosomes were named according to the sorted order of scaffold numbers in the CDC Bethune assembly rather than the chromosome numbers. In the CDC Bethune genome assembly, scaffold numbers and chromosomes numbers are ordered not in the same way, for example, scaffold CP027619.1 is chromosome 1, scaffold CP027620.1 is chromosome 10, and scaffold CP027626.1 is chromosome 2 (NCBI Genome, GCA_000224295.2) [23]. The CDC Bethune chromosome numbers should be used to name the chromosomes in the obtained flax genome assemblies, not the scaffold numbers. Chromosome numbers in the genome assemblies of CDC Bethune and K-3018 v2 correspond to the following chromosome numbers in the genome assemblies of YY5, K-3018 v1, and T397: Chr1–Chr1, Chr2–Chr8, Chr3–Chr9, Chr4–Chr10, Chr5–Chr11, Chr6–Chr12, Chr7–Chr13, Chr8–Chr14, Chr9–Chr15, Chr10–Chr2, Chr11–Chr3, Chr12–Chr4, Chr13–Chr5, Chr14–Chr6, Chr15–Chr7.

BUSCO statistics for the new K-3018 assembly (v2) also revealed slight improvements compared to the previous version (v1): 2232 complete BUSCOs (up from 2228) corresponding to the completeness rise from 95.8% to 96.0%. In the K-3018 v2 genome assembly, 37,081 gene models and 43,116 transcript models were predicted.

**Figure 2:**
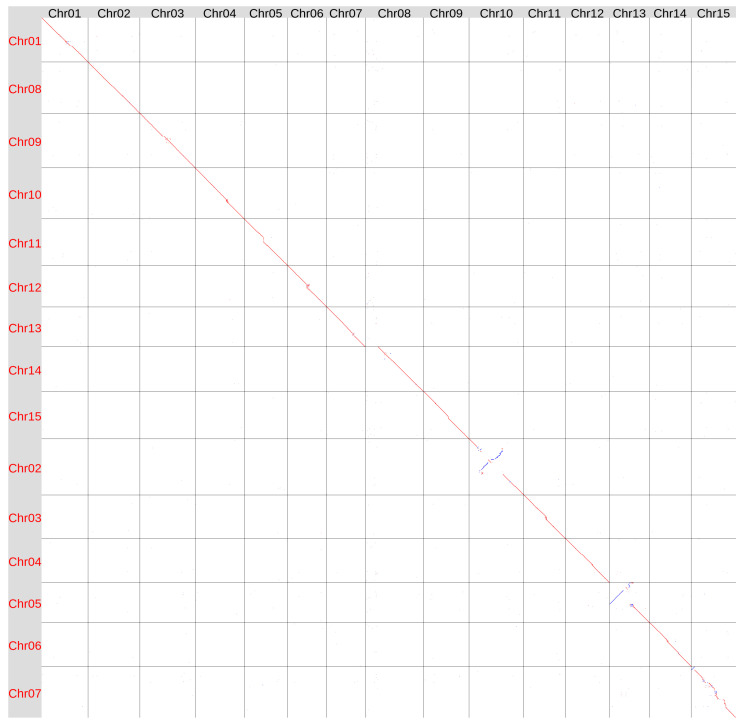
Global alignment of the genome assemblies of flax varieties K-3018 (v2, obtained using Hifiasm ONT v.0.25.0, X axis) and T397 (GCA_051167515, Y axis). Red lines and dots–forward orientation, blue lines and dots—reverse orientation.

We also aligned the Hifiasm ONT-derived genome assembly of variety K-3018 (v2) with the T397 assembly (NCBI Genome, GCA_051167515.1) [31], which is currently the reference in the NCBI Genome database for *L. usitatissimum* (as of 1 November 2025) (Figure 2).

In general, the assemblies were quite similar, but with a few notable exceptions. Two large inversions were detected in Chr10 and Chr13 according to the K-3018 v2 (CDC Bethune) chromosome numeration. Other structural differences included a contiguous variation near the centromeric region of Chr15 and an increased length of Chr08 in the K-3018 v2 genome assembly.

In the T397 genome assembly, Chr05, which corresponds to Chr13 in the K-3018 v2 assembly, contained telomeric repeats only at one end [31] (see also Supplementary Figure S3). In contrast, Chr13 in the K-3018 v2 assembly had telomeric repeats at both ends (Supplementary Figure S2), indicating its accuracy. The analyzed chromosome in the T397 assembly had a half-chromosome-length inversion compared to the K-3018 v2 assembly. The large size of the inversion and the absence of telomeric repeats at the inverted part of the chromosome suggest an error in the T397 genome assembly rather than genetic variation in the genomes of the examined flax varieties.

Another major difference was observed in Chr14 of the T397 assembly, which corresponds to Chr08 in the K-3018 v2 genome assembly. This chromosome was 9.5 Mb shorter in the T397 assembly than in the K-3018 v2 assembly. As previously reported for the K-3018 v1 assembly, this chromosome contains extended telomeric repeats within its internal region [29]. FISH experiments also identified telomeric repeats within the centromeric heterochromatin of one pair of the largest flax chromosomes [34]. Therefore, these internal telomeric repeats are not an artifact. However, in the T397 assembly, this chromosome ends at the location of the internal telomeric repeats in the K-3018 v2 genome assembly (Supplementary Figures S2 and S3, see Chr08 and Chr14, respectively). This suggests that the distal portion of the largest flax chromosome is missing in the T397 assembly.

Other misalignments between the K-3018 v2 and T397 assemblies could be associated with both assembly errors and genetic differences. The T397 genome was initially assembled into 1330 contigs, which were then scaffolded with optical mapping into 20 sequences. They were subsequently curated and aligned to the YY5 assembly, generating 15 chromosome-level scaffolds. In contrast, the K-3018 genome was assembled directly into 17 contigs (with only two remaining gaps) using the ONT sequencing data and Hifiasm ONT algorithm. Telomeric repeats were present at all 30 chromosome ends, indicating high assembly completeness and accuracy.

Unfortunately, another flax genome assembly, which was stated as gapless, will not be available until 30 June 2026 (CNCB, PRJCA037526, https://ngdc.cncb.ac.cn/gwh/Assembly/92583/show, accessed on 1 November 2025).

### 2.2. Genome of the Variety Svyatogor

We sequenced the genome of the flax variety Svyatogor using the ONT platform. As a result, after basecalling with at least Q10 read quality, we generated 27.5 Gb of sequencing data with an N50 of 15.4 kb corresponding to 55× coverage for a 500 Mb genome (46× coverage after filtering for a minimum read length of 5 kb). The Hifiasm ONT algorithm produced a 497.8 Mb genome assembly consisting of 18 contigs with an N50 of 33.3 Mb, and the largest contig was 40.8 Mb. Twelve chromosomes were gapless, while three chromosomes were split into two contigs each. The Svyatogor genome assembly had one more gap than the K-3018 v2 assembly obtained in the present study (three versus two), but it surpassed the T397 assembly [31] in this parameter (three gaps versus five). All assembled chromosomes of variety Svyatogor had telomeric repeats at both ends (Supplementary Figure S4), outperforming the T397 assembly, which had correct telomeric repeats at both ends of 13 chromosomes and at one end of two chromosomes (Chr05 and Chr14).

BUSCO statistics for the Svyatogor genome assembly were 95.9% with 2230 complete BUSCOs. In the Svyatogor genome assembly, 37,207 gene models and 43,303 transcript models were predicted. For the variety Svyatogor, we also obtained 23.8 Gb of Illumina genome sequencing data (48× coverage for a 500 Mb genome). Using these data, a k-mer analysis evaluated the flax genome size to be ~474 Mb (rough estimate), assembly completeness score–99.0%, and QV score–55.3, indicating high completeness and sequence accuracy of the assembled genome. Moreover, we generated the Hi-C data, and the contact map (Supplementary Figure S5) revealed no abnormal interactions.

**Figure 3:**
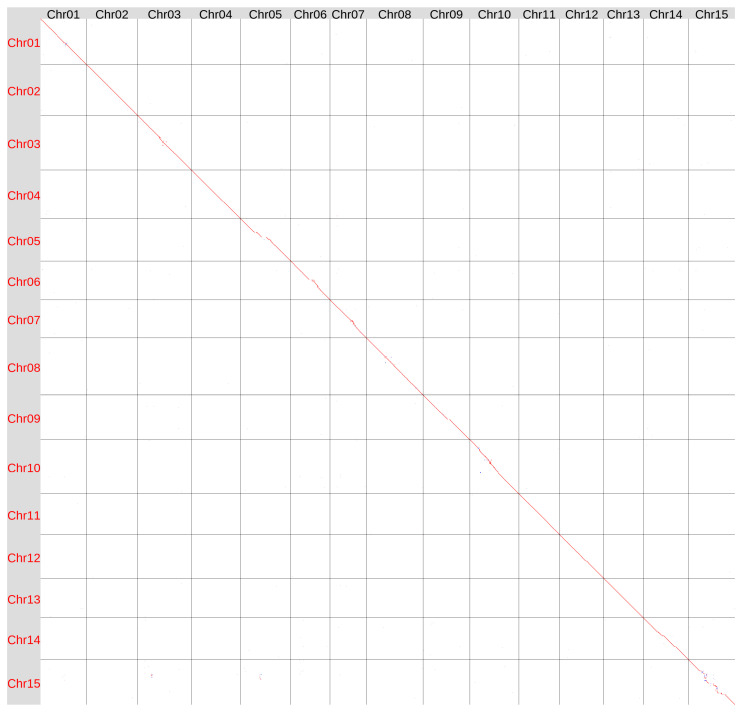
Global alignment of the genome assemblies of flax varieties K-3018 (v2, Y axis) and Svyatogor (X axis) obtained using Hifiasm ONT v.0.25.0. Red lines and dots—forward orientation, blue lines and dots—reverse orientation.

We compared the Hifiasm ONT-derived genomes of the varieties K-3018 and Svyatogor (Figure 3). The assemblies were generally similar, though some differences were observed in the central regions of several chromosomes, which are likely centromeric (especially noticeable on Chr15). These regions are rich in repeats and poor in genes, so differences could occur.

**Figure 4:**
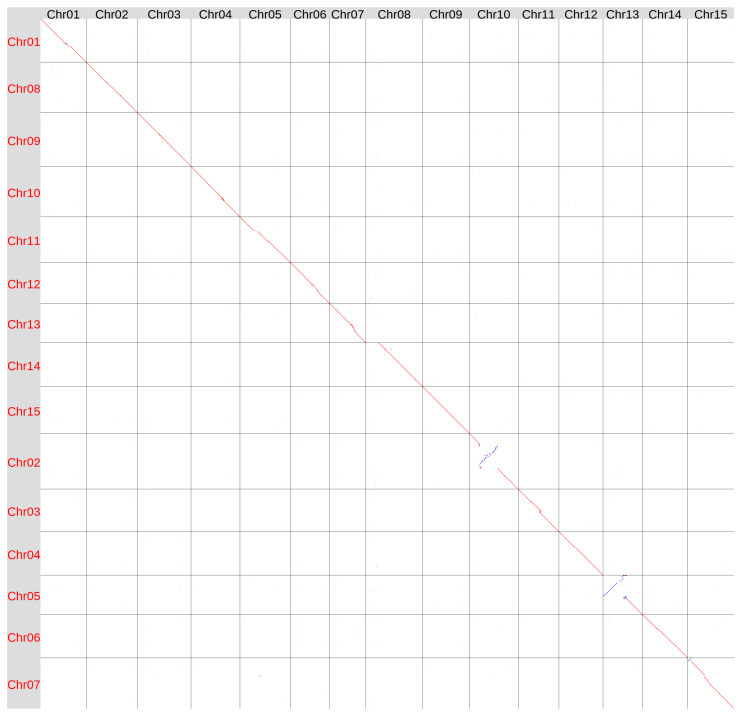
Global alignment of the genome assemblies of flax varieties T397 (GCA_051167515, Y axis) and Svyatogor (X axis). Red lines and dots—forward orientation, blue lines and dots—reverse orientation.

We also compared the genome assemblies of the varieties Svyatogor and T397 (Figure 4). Interestingly, the region near the centromere of Chr15 in Svyatogor was more similar to the corresponding region in T397 than to that in K-3018, suggesting that this region can be highly variable in flax genomes. Chr08 and Chr13 of Svyatogor assembly had the same differences from the corresponding chromosomes of T397 assembly as had Chr08 and Chr13 of K-3018 assembly. This further supported the incorrect/incomplete assembly of these chromosomes in the T397 genome.

Flax genome assemblies of varieties CDC Bethune v2 [23], Atlant [25], YY5 v2 [26], 3896 [27], Heiya 14 [24], Longya 10 [24], Neiya 9 [28], K-1531 [35], which significantly inferior in quality to the genome assemblies of varieties K-3018, Svyatogor, and T397, including decreased genome size, absence of telomeres, tenth and hundreds of gaps, were compared in the previous studies [31,35]. In the present study, we performed comparison of statistics for the latest and best genome assemblies of flax varieties K-3018, Svyatogor, and T397 (Table 1) and created a synteny plot for them (Supplementary Figure S6). Overall, the statistics were quite similar. However, the Svyatogor and K-3018 v2 assemblies slightly outperformed the T397 assembly in all statistics except for the QV score, which was slightly higher for the T397 assembly. The synteny plot (Supplementary Figure S6) showed a number of small translocations, inversions, and/or duplications between the genome assemblies of the three analyzed flax genotypes, which may be related to intervarietal differences. In addition, as we already mentioned above, two substantial (half-chromosome) inversions were present in the T397 assembly within Chr10 and Chr13 (according to the Svyatogor and K-3018 v2 assemblies), thereby differentiating it from the Svyatogor and K-3018 v2 genome assemblies. Because of the inversions’ large sizes and the absence of telomeres at one end of Chr13, these inversions are more likely the T397 assembly errors than intervarietal differences. Additionally, Chr8 (according to the Svyatogor and K-3018 v2 assemblies) in the T397 assembly lacked a significant segment of the chromosome present in the Svyatogor and K-3018 v2 assemblies. This segment is located behind the intrachromosomal telomeric repeat-reach region mentioned above. Chr15 is of particular interest because the highest number of large interchromosomal rearrangements was identified between the three genotypes in this chromosome. Chr15 may differ significantly between flax genotypes; however, additional high-quality flax genome assemblies are needed to clarify this issue.

In our previous study, we compared version 1 (v1) of the K-3018 genome assembly with the YY5 assembly (https://zenodo.org/record/4872894, accessed on 15 August 2025) [26], the best available at the time. We revealed a significant number of mismatches. Based on our analysis of telomeric repeats, we suggested that some of these mismatches were because of the YY5 assembly errors [29]. The present study included the analyses of three genome assemblies: K-3018, Svyatogor, and T397. The results indicated that flax genomes are generally quite similar at the chromosome level, with only a few large-scale differences. Since the YY5 genome assembly likely contains a significant number of misassemblies, comparison of the obtained flax genomes with the YY5 assembly, as was performed in the study by Yadav et al. [31], could lead to misidentification of structural variations. However, several high-quality near-T2T assemblies allow for the proper identification of large-scale genomic alterations in flax genomes.

### 2.3. Progress in the Flax Genome Assembly

In the present study, we obtained two high-quality near-T2T flax genome assemblies with only a few gaps using the ONT simplex sequencing data and ONT-optimized Hifiasm algorithm. All chromosomes in these genome assemblies had pronounced telomeric repeats at the ends, and most chromosomes were assembled from telomere to telomere. Only three chromosomes in Svyatogor and two chromosomes in K-3018 had one gap each. These gaps represent unresolved highly repetitive regions, which can be suggested to be centromeric based on their location. The obtained assemblies surpassed the contiguity and correctness of the T397 genome assembly, which was classified as T2T by Yadav et al. [31] and marked as a reference for *L. usitatissimum* in the NCBI Genome database (as of 1 November 2025, GCA_051167515.1). The T397 genome was assembled using PacBio HiFi and ONT data with optical mapping [31]. In contrast to PacBio HiFi-derived assemblies, flax genomes assembled from R9.4.1 ONT data lacked the sequence accuracy and required polishing with precision Illumina reads [27,35,36]. However, the sequence accuracy of flax genomes assembled from R10.4.1 ONT data and from PacBio HiFi data was already comparable [29]. Using the obtained Illumina reads, we demonstrated the high sequence accuracy of the Svyatogor genome assembly–the QV score was more than 55. Thus, new genome assembly strategies optimized for ONT simplex data and recent algorithmic advances in error correction, including an improved Hifiasm ONT correction module that reliably distinguishes true sequencing errors from phasing differences [37] and avoids the assumption that ONT errors are randomly distributed, have further enhanced the accuracy of ONT-only assemblies. This module has also outperformed alternative machine learning-based correction approaches (such as Herro [38]), enabling the generation of near-error-free genome assemblies. As a result, recent progress in ONT sequencing and bioinformatics has allowed us to produce accurate near-T2T flax genome assemblies from the ONT simplex R10.4.1 reads using Hifiasm ONT without involving PacBio HiFi or UL ONT reads as well as optical maps. These assemblies can compete with and surpass those produced using PacBio HiFi data with either Hi-C scaffolding or UL-ONT hybrid strategies, which rely on costly and difficult-to-generate datasets.

Numerous studies have aimed to develop genetic markers of valuable flax traits [39]. However, most of these studies used the genome assembly of the variety CDC Bethune as a reference. This assembly was initially produced from short Illumina reads [22,23] and then scaffolded to chromosome level. Thus, it was incomplete and insufficiently extensive (only about 316 Mb). New near-T2T-level assemblies of the flax genomes are essential for improving the efficiency of flax genetic research and for evaluating flax genetic diversity at the genome level. They are also crucial for developing approaches for marker-assisted and genomic selection, as well as precise genome editing.

## 3. Materials and Methods

### 3.1. DNA Preparation and Sequencing on the ONT Platform

The high-yield dual-purpose flax variety Svyatogor, which can be used for both seed and stem production and has high resistance to abiotic stresses, was selected for the study.

DNA was extracted from leaves as described in our previous study with the stage of nuclei isolation [27]. The SQK-LSK109 kit (ONT, Oxford, UK) and SQK-LSK114 kit (ONT) were used for DNA library preparation. Genome sequencing was performed using MinION and PromethION sequencers (ONT) with R10.4.1 flow cells (ONT).

### 3.2. Construction and Sequencing of Genomic and Hi-C Libraries on the Illumina Platform

Illumina genomic library for the variety Svyatogor was prepared using the TransNGS Fragmentase DNA Library Prep Kit for Illumina (Transgen, Beijing, China).

Hi-C libraries were prepared for varieties K-3018 and Svyatogor. Chromatin was fixed with formaldehyde using vacuum infiltration. Nuclei were isolated from the fixed leaves. Then, according to a slightly modified protocol [40], restriction with DpnII (New England Biolabs, Ipswich, MA, USA), biotin labeling with Biotin-15-dCTP (Biosan, Novosibirsk, Russia), ligation of DNA fragments with T4 DNA Ligase (New England Biolabs), and phenol-chloroform DNA extraction were performed. Next, the biotin was removed from the unligated ends of the DNA fragments and the Hi-C libraries were prepared using the Qiaseq FX DNA kit (Qiagen, Chatsworth, MD, USA) including the step for enrichment of the biotinylated fragments with Streptavidin Magnetic Beads (New England Biolabs).

The quality and concentration of the libraries were assessed by 2100 Bioanalyzer (Agilent Technologies, Santa Clara, CA, USA) and Qubit 4.0 fluorometer (Thermo Fisher Scientific, Waltham, MA, USA), respectively. The genomic and Hi-C libraries were sequenced on NovaSeq 6000 and NovaSeq X Plus (Illumina, San Diego, CA, USA), respectively, with 150 + 150 nucleotide reads.

### 3.3. Assembly of Flax Genomes

For the genome assembly of variety K-3018, we used the ONT data previously obtained by us (NCBI SRA, SRX26506354). For the genome assembly of variety Svyatogor, we used the ONT reads obtained in the present study.

Basecalling was performed using the Dorado software package (v.1.0.2, https://github.com/nanoporetech/dorado, accessed on 25 August 2025) with a minimum average read quality of Q10. The dna_r10.4.1_e8.2_400bps_sup@v5.2.0 (sup) model was used. The length and quality distribution for the ONT reads was assessed using NanoPlot (v.1.44.1) [41]. Adapter sequences were additionally trimmed using Porechop (v.0.2.4) (https://github.com/rrwick/Porechop, accessed on 25 August 2025) with default parameters. The ONT reads were filtered for a minimum length of 10 kb for the variety K-3018 and 5 kb for the variety Svyatogor and used for genome assembly with the Hifiasm algorithm (v.0.25.0-r726) [37] with parameters optimized for ONT reads (--ont–preset for ONT-based assembly; -l0–disables the purge of duplicates, as recommended for haploid or imbred genome assemblies; --telo-m CCCTAAA–sets telomere motif as CCCTAAA).

The Illumina data for Svyatogor were trimmed using fastp 1.0.1 [42] with default parameters. The T397 Illumina genomic data were downloaded from NCBI SRA (SRX25430339) and treated the same way.

Assembly statistics (including assembly length, number and length of contigs) were assessed using QUAST (v.5.3.0) [43]. Softmasking for genome assemblies was performed with RepeatMasker 4.2.1 [44] using a de novo repeat database constructed with RepeatModeler 2.0.7 [44] using RepBase (2018) [45] and Dfam 3.9 [46]. Structural annotations of genome assemblies were created with Braker3 [47] using the numerous transcriptomic data for the flax variety 3896 [48] and Viridiplantae OrthoDB 11 database [49]. Completeness evaluation of genome assemblies was performed using BUSCO (v.5.8.3) with the eudicots_odb10 database [50] using the genome mode based on miniprot alignment. Consensus quality value (QV) and assembly completeness scores were assessed with Merqury (v1.3) using default parameters for a haploid assembly and k value of 20 [51]. Global alignment of the assemblies was performed using LAST (v.1639) [52] with the --uRY128 option (initial matches between query and reference sequences were searched on ~1/128 of positions in each sequence). The TIDK software package (v.0.2.64) [53] was used for identification of telomeric sequences (the CCCTAAA telomeric motif was searched).

The Hi-C reads were aligned to the corresponding genome assemblies of varieties K-3018 and Svyatogor with BWA-MEM (v.0.7.19) (-5SP parameters) [54]. The identification of ligation sites was performed using pairtools (v.1.1.3) [55]. The contact maps were obtained with Juicer Tools (v.1.22.01) and visualized with Juicebox (v.1.11.08) [56].

Mapping of Illumina reads was performed using BWA-MEM (v.0.7.19) and k-mer distribution analysis was performed using Jellyfish (v.2.3.1) [57] with high count value of histogram set to 100,000. The genome size and duplication rate were assessed using GenomeScope2 with a k-mer length of 20 and ploidy set to 1 (v.2.0.1) [58].

## Figures and Tables

**Table 1:** Statistics of the genome assemblies of flax varieties K-3018, Svyatogor, and T397.

Variety	Assembly Size, Mb	QV Score	Merqury Completeness, %	BUSCO Completeness, %	Number of Gaps	Number of Telomeres
K-3018 v1	489.1	-	-	95.8	9	30
K-3018 v2	491.1	-	-	96.0	2	30
Svyatogor	497.8	55.3	99.0	95.9	3	30
T397	494.9	59.6	96.0	95.8	5	28

## Data Availability

The data generated in this study are available at NCBI under the BioProject accession number PRJNA648016 (linked to BioSamples SAMN44450280 and SAMN52020661).

## Supplementary Materials

The following supporting information can be downloaded at: https://www.mdpi.com/article/10.3390/plants15010151/s1, Figure S1: Hi-C contact map for the flax variety K-3018 v2 genome assembly; Figure S2: Density of telomeric repeats in the flax variety K-3018 v2 genome assembly; Figure S3: Density of telomeric repeats in the flax variety T397 genome assembly; Figure S4: Density of telomeric repeats in the flax variety Svyatogor genome assembly; Figure S5: Hi-C contact map for the flax variety Svyatogor genome assembly; Figure S6: Synteny plot for genome assemblies of flax varieties T397, K-3018 (v2), and Svyatogor.

## References

[1] Sun Y., Shang L., Zhu Q.H., Fan L., Guo L. (2022). Twenty years of plant genome sequencing: Achievements and challenges. Trends Plant Sci..

[2] Espinosa E., Bautista R., Larrosa R., Plata O. (2024). Advancements in long-read genome sequencing technologies and algorithms. Genomics.

[3] Zhang T., Zhou J., Gao W., Jia Y., Wei Y., Wang G. (2022). Complex genome assembly based on long-read sequencing. Brief. Bioinform..

[4] Medhi U., Chaliha C., Singh A., Nath B.K., Kalita E. (2025). Third generation sequencing transforming plant genome research: Current trends and challenges. Gene.

[5] Li H., Durbin R. (2024). Genome assembly in the telomere-to-telomere era. Nat. Rev. Genet..

[6] Diaz-Riano J.I., Duitama J. (2025). Current progress in phased genome assembly from long-read DNA sequencing data. Methods Mol. Biol..

[7] Mahmoud M., Agustinho D.P., Sedlazeck F.J. (2025). A Hitchhiker’s guide to long-read genomic analysis. Genome Res..

[8] Bernal-Gallardo J.J., de Folter S. (2024). Plant genome information facilitates plant functional genomics. Planta.

[9] Garg S., Nain P., Kumar A., Joshi S., Punetha H., Sharma P.K., Siddiqui S., Alshaharni M.O., Algopishi U.B., Mittal A. (2024). Next generation plant biostimulants & genome sequencing strategies for sustainable agriculture development. Front. Microbiol..

[10] Dmitriev A.A., Pushkova E.N., Melnikova N.V. (2022). Plant genome sequencing: Modern technologies and novel opportunities for breeding. Mol. Biol..

[11] Schreiber M., Jayakodi M., Stein N., Mascher M. (2024). Plant pangenomes for crop improvement, biodiversity and evolution. Nat. Rev. Genet..

[12] Kumar R., Das S.P., Choudhury B.U., Kumar A., Prakash N.R., Verma R., Chakraborti M., Devi A.G., Bhattacharjee B., Das R. (2024). Advances in genomic tools for plant breeding: Harnessing DNA molecular markers, genomic selection, and genome editing. Biol. Res..

[13] Jayakodi M., Shim H., Mascher M. (2025). What are we learning from plant pangenomes?. Annu. Rev. Plant Biol..

[14] Naithani S., Deng C.H., Sahu S.K., Jaiswal P. (2023). Exploring pan-genomes: An overview of resources and tools for unraveling structure, function, and evolution of crop genes and genomes. Biomolecules.

[15] Garg V., Bohra A., Mascher M., Spannagl M., Xu X., Bevan M.W., Bennetzen J.L., Varshney R.K. (2024). Unlocking plant genetics with telomere-to-telomere genome assemblies. Nat. Genet..

[16] Tse T.J., Guo Y., Shim Y.Y., Purdy S.K., Kim J.H., Cho J.Y., Alcorn J., Reaney M.J.T. (2023). Availability of bioactive flax lignan from foods and supplements. Crit. Rev. Food Sci. Nutr..

[17] Gao Z., Cao Q., Deng Z. (2024). Unveiling the power of flax lignans: From plant biosynthesis to human health benefits. Nutrients.

[18] Stepien A.E., Trojniak J., Tabarkiewicz J. (2025). Anti-oxidant and anti-cancer properties of flaxseed. Int. J. Mol. Sci..

[19] Campos J.R., Severino P., Ferreira C.S., Zielinska A., Santini A., Souto S.B., Souto E.B. (2019). Linseed essential oil—source of lipids as active ingredients for pharmaceuticals and nutraceuticals. Curr. Med. Chem..

[20] Kezimana P., Dmitriev A.A., Kudryavtseva A.V., Romanova E.V., Melnikova N.V. (2018). Secoisolariciresinol diglucoside of flaxseed and its metabolites: biosynthesis and potential for nutraceuticals. Front. Genet..

[21] Goudenhooft C., Bourmaud A., Baley C. (2019). Flax (*Linum usitatissimum* L.) Fibers for composite reinforcement: Exploring the link between plant growth, cell walls development, and fiber properties. Front. Plant Sci..

[22] Wang Z., Hobson N., Galindo L., Zhu S., Shi D., McDill J., Yang L., Hawkins S., Neutelings G., Datla R. (2012). The genome of flax (*Linum usitatissimum*) assembled de novo from short shotgun sequence reads. Plant J..

[23] You F.M., Xiao J., Li P., Yao Z., Jia G., He L., Zhu T., Luo M.C., Wang X., Deyholos M.K. (2018). Chromosome-scale pseudomolecules refined by optical, physical and genetic maps in flax. Plant J..

[24] Zhang J., Qi Y., Wang L., Wang L., Yan X., Dang Z., Li W., Zhao W., Pei X., Li X. (2020). Genomic comparison and population diversity analysis provide insights into the domestication and improvement of flax. Iscience.

[25] Dmitriev A.A., Pushkova E.N., Novakovskiy R.O., Beniaminov A.D., Rozhmina T.A., Zhuchenko A.A., Bolsheva N.L., Muravenko O.V., Povkhova L.V., Dvorianinova E.M. (2021). Genome sequencing of fiber flax cultivar Atlant using Oxford Nanopore and Illumina platforms. Front. Genet..

[26] Sa R., Yi L., Siqin B., An M., Bao H., Song X., Wang S., Li Z., Zhang Z., Hazaisi H. (2021). Chromosome-level genome assembly and annotation of the fiber flax (*Linum usitatissimum*) genome. Front. Genet..

[27] Dvorianinova E.M., Bolsheva N.L., Pushkova E.N., Rozhmina T.A., Zhuchenko A.A., Novakovskiy R.O., Povkhova L.V., Sigova E.A., Zhernova D.A., Borkhert E.V. (2022). Isolating *Linum usitatissimum* L. nuclear DNA enabled assembling high-quality genome. Int. J. Mol. Sci..

[28] Zhao X., Yi L., Zuo Y., Gao F., Cheng Y., Zhang H., Zhou Y., Jia X., Su S., Zhang D. (2023). High-quality genome assembly and genome-wide association study of male sterility provide resources for flax improvement. Plants.

[29] Arkhipov A.A., Pushkova E.N., Bolsheva N.L., Rozhmina T.A., Borkhert E.V., Zhernova D.A., Rybakova T.Y., Barsukov N.M., Moskalenko O.D., Sigova E.A. (2024). Nanopore data-driven chromosome-level assembly of flax genome. Plants.

[30] Lu J., Wu H., Wang F., Li J., Wang Y., Zhao Q., Wang Y., Wang X., Lei X., Sun R. (2025). Telomere to telomere flax (*Linum usitatissimum* L.) genome assembly unlocks insights beyond fatty acid metabolism pathways. Hortic. Res..

[31] Yadav H.K., Singh N., Singh B., Kaur V., Sawant S.V. (2025). Telomere-to-telomere genome assembly of linseed (*Linum usitatissimum* L.) for functional genomics and accelerated genetic improvement. Plant Biotechnol. J..

[32] Pushkova E.N., Borkhert E.V., Novakovskiy R.O., Dvorianinova E.M., Rozhmina T.A., Zhuchenko A.A., Zhernova D.A., Turba A.A., Yablokov A.G., Sigova E.A. (2023). Selection of flax genotypes for pan-genomic studies by sequencing tagmentation-based transcriptome libraries. Plants.

[33] Cloutier S., Ragupathy R., Miranda E., Radovanovic N., Reimer E., Walichnowski A., Ward K., Rowland G., Duguid S., Banik M. (2012). Integrated consensus genetic and physical maps of flax (*Linum usitatissimum* L.). Theor. Appl. Genet..

[34] Bolsheva N.L., Semenova O.Y., Muravenko O., Nosova I.V., Popov K., Zelenin A.V. (2005). Localization of telomere sequences in chromosomes of two flax species. Biol. Membr..

[35] Dvorianinova E.M., Pushkova E.N., Bolsheva N.L., Borkhert E.V., Rozhmina T.A., Zhernova D.A., Novakovskiy R.O., Turba A.A., Sigova E.A., Melnikova N.V. (2023). Genome of *Linum usitatissimum* convar. *crepitans* expands the view on the section *Linum*. Front. Genet..

[36] Dvorianinova E.M., Pushkova E.N., Bolsheva N.L., Rozhmina T.A., Zhernova D.A., Sigova E.A., Borkhert E.V., Melnikova N.V., Dmitriev A.A. (2023). Improving genome assembly of flax line 3896 with high-precision Illumina reads. Russ. J. Genet..

[37] Cheng H., Qu H., McKenzie S., Lawrence K.R., Windsor R., Vella M., Park P.J., Li H. (2025). Efficient near telomere-to-telomere assembly of Nanopore simplex reads. bioRxiv.

[38] Stanojević D., Lin D., Nurk S., Florez de Sessions P., Šikić M. (2024). Telomere-to-telomere phased genome assembly using HERRO-corrected simplex Nanopore reads. bioRxiv.

[39] Zhernova D.A., Pushkova E.N., Rozhmina T.A., Borkhert E.V., Arkhipov A.A., Sigova E.A., Dvorianinova E.M., Dmitriev A.A., Melnikova N.V. (2024). History and prospects of flax genetic markers. Front. Plant Sci..

[40] Zelenka T., Spilianakis C. (2021). HiChIP and Hi-C protocol optimized for primary murine T cells. Methods Protoc..

[41] De Coster W., Rademakers R. (2023). NanoPack2: Population-scale evaluation of long-read sequencing data. Bioinformatics.

[42] Chen S., Zhou Y., Chen Y., Gu J. (2018). fastp: An ultra-fast all-in-one FASTQ preprocessor. Bioinformatics.

[43] Gurevich A., Saveliev V., Vyahhi N., Tesler G. (2013). QUAST: Quality assessment tool for genome assemblies. Bioinformatics.

[44] Flynn J.M., Hubley R., Goubert C., Rosen J., Clark A.G., Feschotte C., Smit A.F. (2020). RepeatModeler2 for automated genomic discovery of transposable element families. Proc. Natl. Acad. Sci. USA.

[45] Bao W., Kojima K.K., Kohany O. (2015). Repbase Update, a database of repetitive elements in eukaryotic genomes. Mob. DNA.

[46] Storer J., Hubley R., Rosen J., Wheeler T.J., Smit A.F. (2021). The Dfam community resource of transposable element families, sequence models, and genome annotations. Mob. DNA.

[47] Gabriel L., Bruna T., Hoff K.J., Ebel M., Lomsadze A., Borodovsky M., Stanke M. (2024). BRAKER3: Fully automated genome annotation using RNA-seq and protein evidence with GeneMark-ETP, AUGUSTUS and TSEBRA. Genome Res..

[48] Zhernova D.A., Arkhipov A.A., Rozhmina T.A., Zhuchenko A.A., Bolsheva N.L., Sigova E.A., Dvorianinova E.M., Borkhert E.V., Pushkova E.N., Melnikova N.V. (2025). Transcriptome map and genome annotation of flax line 3896. Front. Plant Sci..

[49] Kuznetsov D., Tegenfeldt F., Manni M., Seppey M., Berkeley M., Kriventseva E.V., Zdobnov E.M. (2023). OrthoDB v11: Annotation of orthologs in the widest sampling of organismal diversity. Nucleic Acids Res..

[50] Manni M., Berkeley M.R., Seppey M., Simao F.A., Zdobnov E.M. (2021). BUSCO update: Novel and streamlined workflows along with broader and deeper phylogenetic coverage for scoring of eukaryotic, prokaryotic, and viral genomes. Mol. Biol. Evol..

[51] Rhie A., Walenz B.P., Koren S., Phillippy A.M. (2020). Merqury: Reference-free quality, completeness, and phasing assessment for genome assemblies. Genome Biol..

[52] Kielbasa S.M., Wan R., Sato K., Horton P., Frith M.C. (2011). Adaptive seeds tame genomic sequence comparison. Genome Res..

[53] Brown M.R., Manuel Gonzalez de La Rosa P., Blaxter M. (2025). tidk: A toolkit to rapidly identify telomeric repeats from genomic datasets. Bioinformatics.

[54] Li H. (2013). Aligning sequence reads, clone sequences and assembly contigs with BWA-MEM. arXiv.

[55] Open2C, Abdennur N., Fudenberg G., Flyamer I.M., Galitsyna A.A., Goloborodko A., Imakaev M., Venev S.V. (2023). Pairtools: From sequencing data to chromosome contacts. bioRxiv.

[56] Durand N.C., Shamim M.S., Machol I., Rao S.S., Huntley M.H., Lander E.S., Aiden E.L. (2016). Juicer provides a one-click system for analyzing loop-resolution Hi-C experiments. Cell Syst..

[57] Marcais G., Kingsford C. (2011). A fast, lock-free approach for efficient parallel counting of occurrences of k-mers. Bioinformatics.

[58] Ranallo-Benavidez T.R., Jaron K.S., Schatz M.C. (2020). GenomeScope 2.0 and Smudgeplot for reference-free profiling of polyploid genomes. Nat. Commun..

