# Supplementary material for: Progress in Flax Genome Assembly from Nanopore Sequencing Data
Source: Plants (Basel). 2026 Jan 4;15(1):151. doi: 10.3390/plants15010151 (PMC12787752; doi:10.3390/plants15010151)
Supplement: Supplementary file 1 [file plants-15-00151-s001.zip › Fig_S2_K-3018_v2_telomeres.pdf]

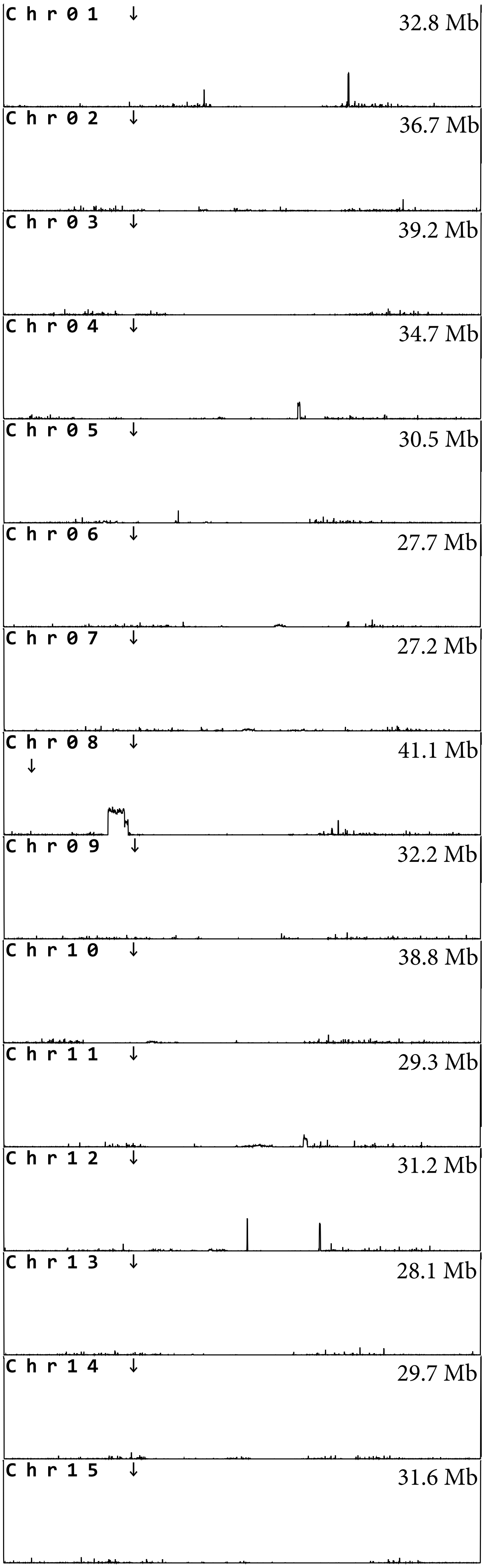

Figure S2: Density of telomeric repeats in the flax variety K-3018 genome assembly. Density of CCCTAAA motif was calculated using 10-kb sliding windows. Each graph is scaled to the maximum motif density value on the Y-axis, and to the chromosome length on the X-axis. Distinct peaks marking telomeres are visible at the respective chromosome ends.
