# Supplementary figures and images for: Progress in Flax Genome Assembly from Nanopore Sequencing Data
Source: Plants (Basel). 2026 Jan 4;15(1):151. doi: 10.3390/plants15010151 (PMC12787752; doi:10.3390/plants15010151)

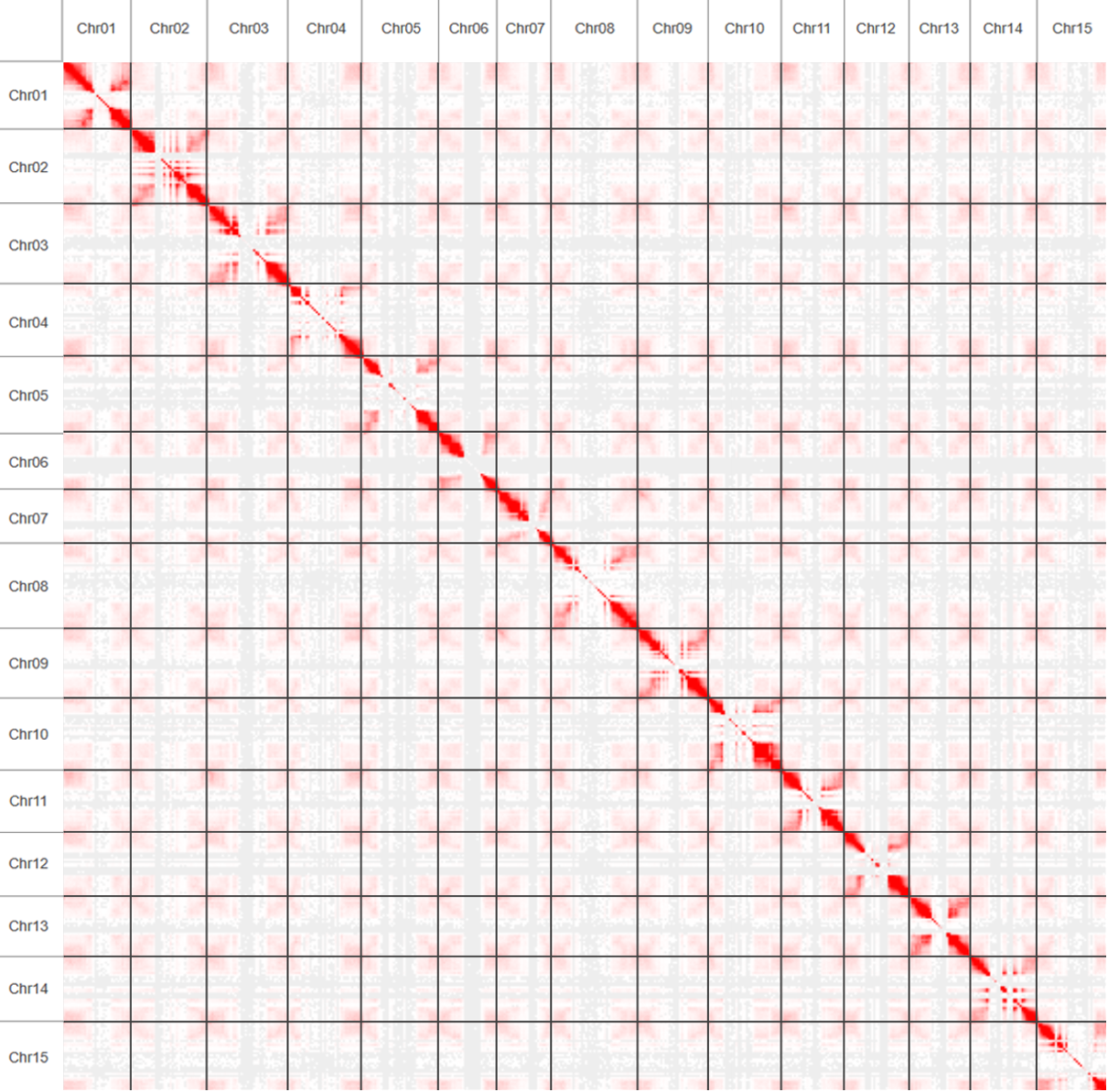

Supplement: Supplementary file 1 [file plants-15-00151-s001.zip › Fig_S5_Svyatogor_Hi-C.png]

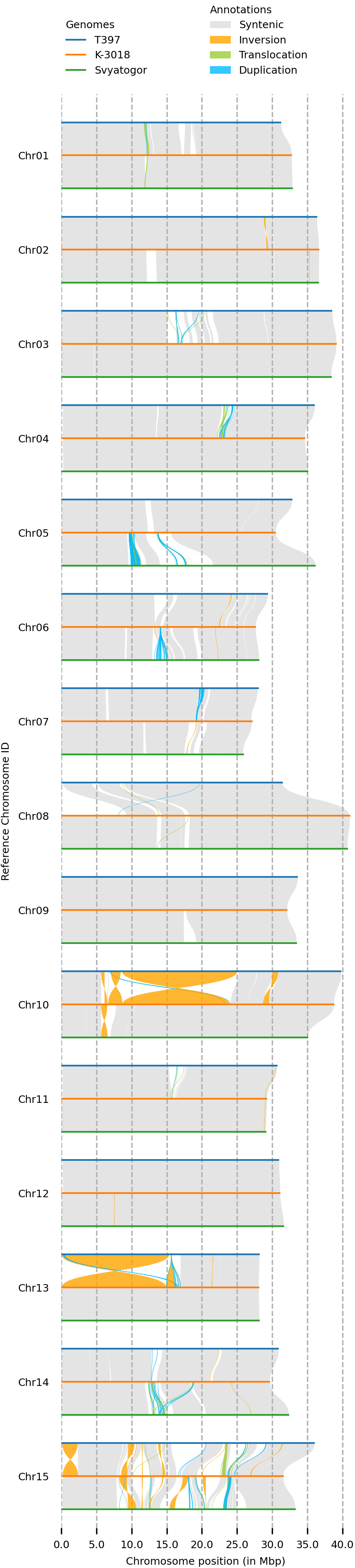

Supplement: Supplementary file 1 [file plants-15-00151-s001.zip › Fig_S6_Synteny_K-3018_Svyatogor_T397.png]

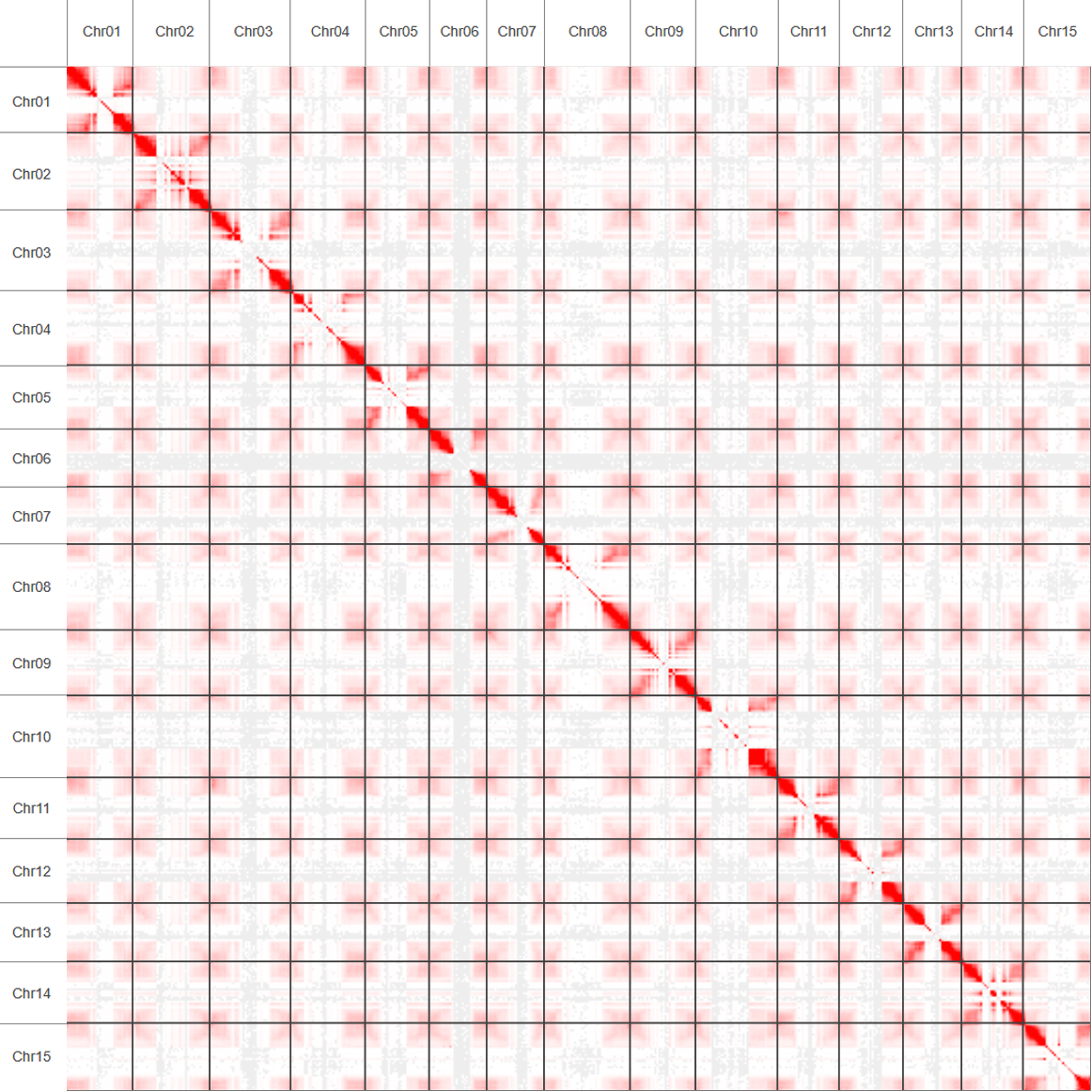

Supplement: Supplementary file 1 [file plants-15-00151-s001.zip › Fig_S1_K-3018_v2_Hi-C.png]
